# A Digital Compressed Sensing-Based Energy-Efficient Single-Spot Bluetooth ECG Node

**DOI:** 10.1155/2018/2687389

**Published:** 2018-01-11

**Authors:** Kan Luo, Zhipeng Cai, Keqin Du, Fumin Zou, Xiangyu Zhang, Jianqing Li

**Affiliations:** ^1^Fujian Key Laboratory of Automotive Electronics and Electric Drive and School of Information Science and Engineering, Fujian University of Technology, Fuzhou 350118, China; ^2^School of Instrument Science and Engineering, Southeast University, Nanjing 210096, China

## Abstract

Energy efficiency is still the obstacle for long-term real-time wireless ECG monitoring. In this paper, a digital compressed sensing- (CS-) based single-spot Bluetooth ECG node is proposed to deal with the challenge in wireless ECG application. A periodic sleep/wake-up scheme and a CS-based compression algorithm are implemented in a node, which consists of ultra-low-power analog front-end, microcontroller, Bluetooth 4.0 communication module, and so forth. The efficiency improvement and the node's specifics are evidenced by the experiments using the ECG signals sampled by the proposed node under daily activities of lay, sit, stand, walk, and run. Under using sparse binary matrix (SBM), block sparse Bayesian learning (BSBL) method, and discrete cosine transform (DCT) basis, all ECG signals were essentially undistorted recovered with root-mean-square differences (PRDs) which are less than 6%. The proposed sleep/wake-up scheme and data compression can reduce the airtime over energy-hungry wireless links, the energy consumption of proposed node is 6.53 mJ, and the energy consumption of radio decreases 77.37%. Moreover, the energy consumption increase caused by CS code execution is negligible, which is 1.3% of the total energy consumption.

## 1. Introduction

Cardiovascular diseases (CVDs) are a major threat to human health. According to the report of WTO, about 17.5 million people died of heart disease every year around the world [1]. Furthermore, the cost for CVD-related treatment is substantial, which is estimated approximately to be 3.8 trillion U.S. dollars in all low- and middle-income countries during 2011–2025 [2]. The situation will be even more severe due to the increasing aging population. Many of CVD-related deaths and associated economic losses can be avoided if the diseases have been early prevented, diagnosed, and treated.

Electrocardiogram (ECG) can give an insight of heart status for diagnosis of CVDs, and it is a standard medical examination in clinical practices nowadays [3]. However, there still exist some limitations of traditional ECG instruments for early diagnosis of CVDs, such as in-hospital short-term examination, huge in volume, inconvenient movement, wired connection, and low autonomy [4]. They cannot satisfy the requirement for long-term, real-time monitoring and feedback in mobile scenarios as most of the early stage CVDs are accidental during daily activity and out of a hospital. Development of inexpensive continuous ambulatory ECG monitoring device becomes a challenge in real-time, long-term, and convenient ECG monitoring.

In recent years, with the rapid development of wireless body sensor networks (WBSNs) and wearable techniques, lots of WBSN-enabled ambulatory ECG monitoring devices have been developed [1, 5, 6]. They could be seamlessly integrated into patients' life for heart status monitoring, providing early warning to avoid accidental adverse cardiovascular events. However, most of such existing devices need to be further improved to advance energy efficiency, which is the major obstacle for long-term wireless ECG monitoring.

Different studies for the energy-efficient node have been investigated from various aspects, including hardware, communication protocol, scheme, coding technology, and data compression methods. Low-power hardware can directly reduce the energy consumption. Yazicioglu et al. [7] proposed mixed-signal design approaches to reduce overall power dissipation of the biopotential sensor node. Tsai et al. [8] fabricated a low-power analog front-end IC, using a 0.18 *μ*m CMOS standard, for effectively wireless ECG acquisition. Energy-efficient protocol or strategy can also prolong the node's lifetime. Nemati employed ANT protocol as a low-data-rate wireless module to reduce the power consumption of a wireless capacitive ECG node. Yan et al. [9] proposed a distance-based energy-efficient data strategy, which lowered the transmission power in both the sensor node level and the network level. Also, considering communication consumes more than 65% of the total energy [10], lots of coding or compression approaches were proposed to optimize the radio energy consumption. A proper compression technology can reduce the amount of the transmitted data, thus improving the node's energy efficiency. As most of the high-performance compression schemes are not compatible with resource-constraint node [11, 12], low computational complexity coding or compression methods were investigated, such as the Walsh transform based variable-word-length coding [13], nonuniform sampling-based dynamic compression [1], and compressed sensing- (CS-) based method [14–16]. Particularly, as a novel sampling paradigm, CS combines the sampling and compression into one step. It efficiently collects signal following the “information rate” instead of the traditional “Nyquist rate” [17–19]. The reported CS-enabled wireless ECG monitoring [14, 15, 20, 21] shows the advantages of CS method in low complexity, low-cost, and energy efficiency.

Existing literature has shown that achieving truly energy-efficient wireless ECG node requires not only ultra-low-power devices and advanced communication protocols but also proper data compression technologies. Although these existing studies explored power saving methods from different aspects, few of them gave out a whole energy-efficient node scheme.

Motivated by these challenges, a digital CS-based single-spot Bluetooth ECG node was designed and implemented. To achieve long-term wireless ECG monitoring, ultra-low-power hardware, such as Bluetooth Low Energy (BLE) communication protocol module, analog front-end (AFE) chips AD8232 and MSP430F1611, were considered in the node design. Meanwhile, the CS-based compression and the periodic sleep/wake-up scheme, which aims at minimizing energy consumption of data communication, were proposed. Particularly, the tiny node taking advantages of low computation complexity, a sparse binary measurement matrix, was implanted in to realize the CS-based compression. The compression technology and sleep strategy can not only reduce the amount of the transmitted data but also decrease the airtime over energy-hungry wireless links, thus improving the node's energy efficiency. To identify the specifics of the prototype node, the experiments were carried out. Also, the results were compared with three commercial nodes.

The paper is organized as follows.  Section 2 elaborated the design of the proposed wireless ECG node. The details of digital CS-based compression and dual-clock source-based periodic sleep/wake-up scheme are illustrated in this section. In Section 3, experimental setups were introduced, including the experimental data and the evaluation indices.  Section 4 demonstrated the experimental results over optimal parameters of CS compression, collected ECG signals under daily activities, energy consumption, and so forth.  Section 5 discussed the advantages and the potential limitation of our node. The summarization of this study was presented in Section 6.

## 2. Designed Wireless ECG Node

In a wireless ECG node, the limited battery power is mainly consumed by three components: sensing, computing, and communication. High-energy consumption hardware, poor power management, and direct transmission of ECG data are energy wasting. To reduce the energy consumption, the proposed node optimized for hardware design, a periodic sleep/wake-up scheme, and CS-based data compression. The objectives of the node are summarized as follows:

1. Ultra-low-power hardware: to save energy in circuit
2. Periodic sleep/wake-up strategy: to reduce airtime over the power-hungry wireless link
3. Compression algorithm with high compression ratio and good recovered quality: to decrease transmission data and guarantee nondistortion diagnosis
4. Compact and low complexity: to realize the compression algorithm on resource-constraint sensing nodes
5. Real-time: to provide online wireless heart status monitoring

### 2.1. Hardware Design

**Figure 1:**
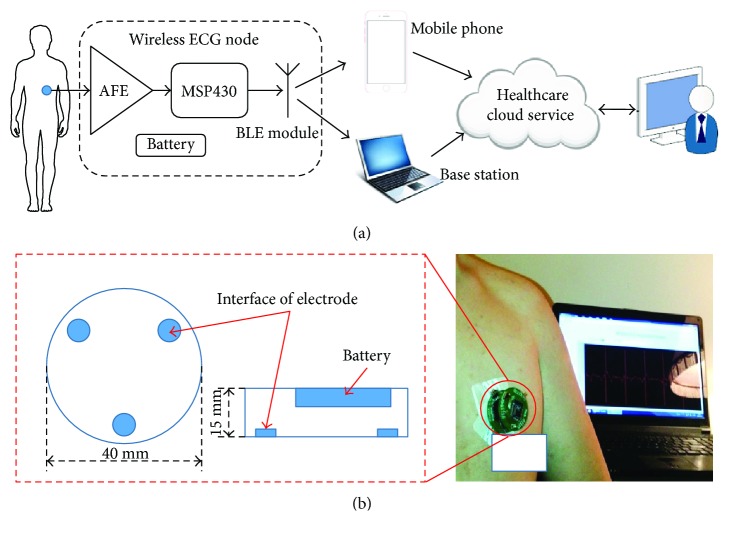
Hardware framework of proposed wireless ECG node. (a) System framework (b) structure, and prototype of the proposed node.

The hardware framework of proposed single-spot wireless ECG node is described in Figure 1. The system is powered by one 3 V CR2032 button battery. Through three electrodes, the ECG signal was obtained and transmitted to an AFE for amplifying and filtering. Subsequently, the preprocessed signal is converted into digital signal by the 12-Bit integrated Analog-to-Digital Converter (ADC) module of MSP430 at 200 Hz. After the compression processing, the data is transmitted to a healthcare cloud server-connected gateway (a mobile phone or a base station) through a BLE transceiver. The valuable medical information will be extracted from the reconstructed signals for the authorized doctor, patient, or medical institution, and so forth. Here, AD8232 is chosen as the AFE. AD8232 is a fully integrated single-lead ECG AFE, which has low supply current (170 *μ*A) and high common-mode rejection ratio (80 dB), and also includes multiple amplifiers and filters. The single chip can easily realize traditional complex ECG preprocessing circuit design. The ECG signal is essentially quasi-periodic nonstationary with a small amplitude (0.01~5 mV) and low frequency (0.05~100 Hz) [22]. The gain of AD8232 was fixed as 500, and the frequency band of the filter was set as 0.5~35 Hz. MSP430F1611 was adopted as core processor. The large Flash (48 kB) and RAM (10 kB) ensure that the node has enough resources for algorithm implementation. Also, there two crystal oscillators, 8 MHz and 11.0592 KHz, are set up as master and second clock sources, which provide the conditions for the node to work at high- or low-speed operation modes. Furthermore, as a bridge between the node and gateway, the transceiver HM-11 provides a short-range (10 m), high-throughput (up to 1 Mb/s data rate) BLE wireless data communication.

The final manufactured node can be found in Figure 1(b). It is a circular structure. The diameter and height are 40 mm and 15 mm, respectively. Moreover, the overall weight (including the battery) is 30 g. Three integrated interfaces of electrodes are uniformly distributed on the nodes at 120 degrees, which are used to connect the standard Ag/AgCl electrodes. In practical, the node can firmly stick to the skin.

### 2.2. Dual-Clock Source-Based Periodic Sleep/Wake-up Scheme

**Figure 2:**
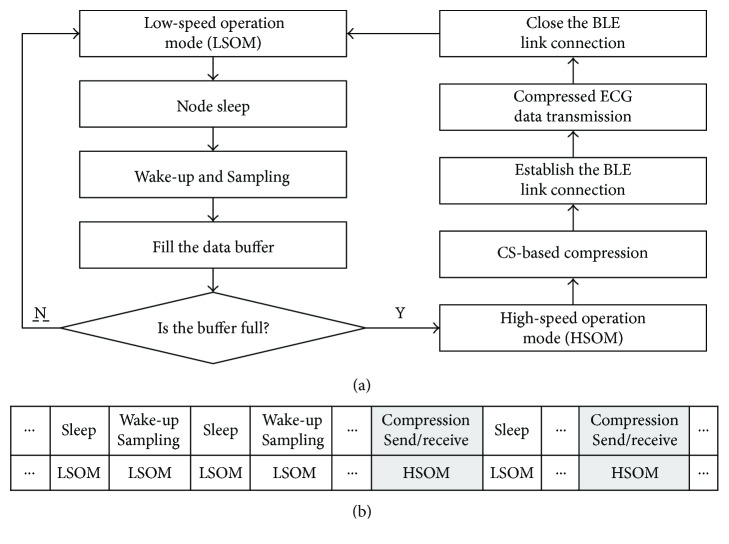
Dual-clock source-based periodic sleep/wake-up scheme. (a) Flowchart of the operation modes. (b) Sequence diagram of the operation modes.

The proposed dual-clock source-based periodic sleep/wake-up scheme is shown in Figure 2. The basic idea of the proposed scheme is that resource allocation is in terms of event slots. Since energy consumption is proportional to clock frequency [9], the node works at high-speed operation mode (HSOM) with the master clock during tasks of data compression and transmission and at low-speed operation mode (LSOM) with the secondary clock in tasks of sampling. Meanwhile, the node periodic sleeps and wakes up in LSOM. The flowchart in Figure 2(a) shows the transformation between the two operation modes. That is, during sampling task, the node is in LSOM, and it keeps sleeping during idle statue and immediately wakes up when periodic sampling event triggered, then followed by filling and data buffer checking. If the data buffer is full, the node moves into HSOM; otherwise, the node keeps in LSOM and repeat sampling. There is no sleeping in HSOM; the node runs at full speed for CS-based data compression and data transmission. After data transmission, the node moves back to LSOM. The sequence diagram of the operation mode is illustrated in Figure 2(b). The node is working in LSOM with sleep/wake-up at most of the time that guarantees energy saving.

### 2.3. Implementation of CS-Based ECG Compression

Recently, the compressed sensing theory was proposed [17–19]. It has broken the traditional sampling rule. The basic theory of the CS is that the sparse signals can be reconstructed from incoherent random measurements [23, 24]. The formal definition of CS is **Y** = **Φ***X*, where *X* is the *N*-dimensional input signal, Φ is *M × N* measurement matrix (*M* < *N*), which represents dimensionality reduction, and **Y** is the collected *M*-length compressed vector. Using CS can reduce the wirelessly transmitted data during the signal acquisition.

There are three critical aspects in ECG compression: the sparsity of the ECG signal, the measurement matrix, and the recovery algorithm. ECG is sparse or sparse in some domains has already been proven in previous studies [14, 15, 20, 21].

**Figure 3:**
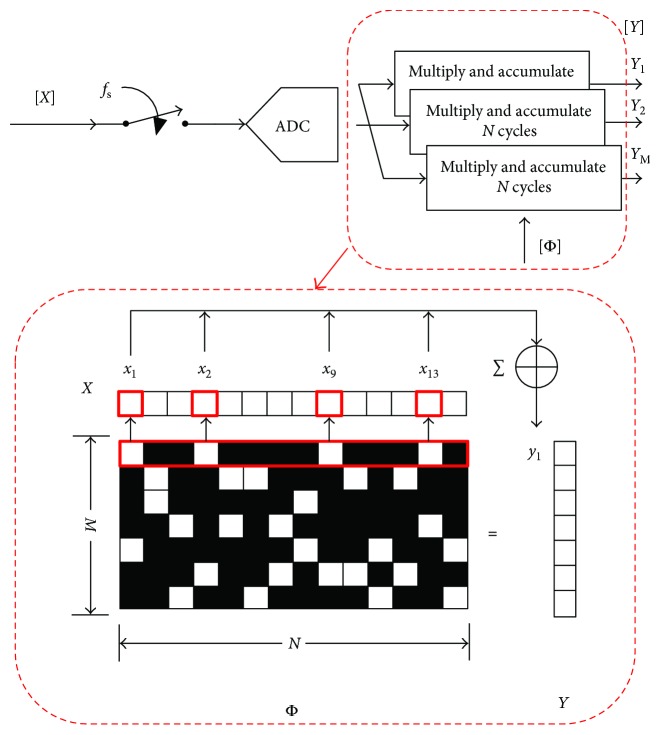
Scheme of digital CS compression on node.

The essential content of digital CS-based compression can be summarized as using measurement matrix multiplication, which includes the multiplication and accumulation, to shorten the signal length. The scheme of the compression is shown in Figure 3. The ECG signal *X* is converted to digital signal following the “Nyquist” sampling rate *f*_*s*_, and then the measurement matrix **Φ** multiplies *X* to get compressed signal **Y**. The digital CS combined the traditional “Nyquist” sampling and the principle of CS signal acquisition; it is suitable for the scenario of sparsity signal compression when ADC can provide enough sampling rate.

The measurement matrix is the key to CS-based compression. The parameters of measurement matrix, which include the bit precision of coefficients, the type of random distribution, and the structure of the matrix, directly affect the compression efficiency and the computation complexity. Commonly used measurement matrices include Gaussian distribution matrix, Bernoulli distribution matrix, and uniform distribution matrix. The bit precision of matrix coefficient ranges from 1 to 64. The Gaussian, Bernoulli, or uniform distribution matrices with high bit coefficient precision are costly because they are difficult to generate and store in a resource constraint nodes; moreover, they bring more computation and higher energy consumption. In comparison, sparse binary matrix (SBM) is more suitably used in a resource-constraint node as measurement matrix [17–19].

SBM has characteristics of sparsity, binary, and incoherent, which is described as *M* × *N* sparse matrix with *K* as one of the elements in each column (*K* < <*M*). **ϕ**_*ij*_ (**ϕ**_*ij*_ ∈ [0,1]) represents the element of **Φ**. In each column of **Φ**, the number of one is far less than the number of zero, and the locations of the one element are random and satisfy the condition of independent identically distributed (i.i.d.).

The implementation of SBM-based compression is marked by the red dashed line in Figure 3. Let *P*_*i*_ = {*p*_*i*_^1^, *p*_*i*_^2^,…, *p*_*i*_^*j*^} denote the locations of one entry in the *i*th row of **Φ**, and the compressed measurement results *y*^(*i*)^ can be updated by (1) without the multiplier.

(1) $$\begin{array}{c} y_{i}=\sum x_{p_{i}^{j}}. \end{array}$$

Figure 3 shows an example, the length of *X* is 14, and **Φ** is a 7 × 14 SBM with *K* = 2. The locations of one entry in the first row are {1, 4, 9, and 13}, then *y*_1_ = *x*_1_ + *x*_4_ + *x*_9_ + *x*_13_. Repeating processing of each row of **Φ**, then the compressed data **Y** is achieved.

### 2.4. Implementation of ECG Reconstruction

The high signal quality recovery algorithm is the key to ECG reconstruction implementation. It will run on a powerful computing gateway or cloud healthcare servers. Assume ***α*** is a sparse vector and **Ψ** is a sparse basis, signal *X* can expand as *X* = Ψ**α**; then the compressed signal is **Y** = **Φ**Ψ**α**. According to the CS theory, it is highly possible to get exact ***α*** when the measurement matrix and the sparsity of the signal satisfy the restricted isometry property [23, 24]. In the proposed framework, lots of excellent algorithms, such as the basis pursuit denoising (BPDN) model, smoothed l0 algorithm, orthogonal matching pursuit, and block sparse Bayesian learning (BSBL), can be used for CS recovery [25–28]. For example, using BSBL and discrete cosine transform (DCT) basis for wireless CS compressed ECG recovery. If the measurement matrix **Φ** and DCT basis Ψ are known, the solution $\hat{\alpha }$ is output after **Φ**Ψ, and received **Y** is fed in the BSBL algorithm; then the recovered ECG is $\hat{X}=\Psi \hat{\alpha }$ when $\hat{\alpha }$ is got. The comparison results of different recovery algorithms are demonstrated in the Results section.

## 3. Experiment Designs

The proposed node was evaluated in data compression and energy consumption. The experiment setups are similar to our previous work [1]. The testbed consists of the proposed node, a data acquisition (DAQ) card (National Instrument USB6009, 14 bits, maximum 48 K sampling rate), and a laptop (Intel i7 4720qm, 8G RAM, Bluetooth 4.0) with Matlab 2016a and LabVIEW 2013. The DAQ card is connected to the laptop by USB cables. The node is wirelessly connected to the base station (the laptop). The measurement matrix is SBM, and the length of ECG signal *N* is 512 in all experiments.

**Figure 4:**
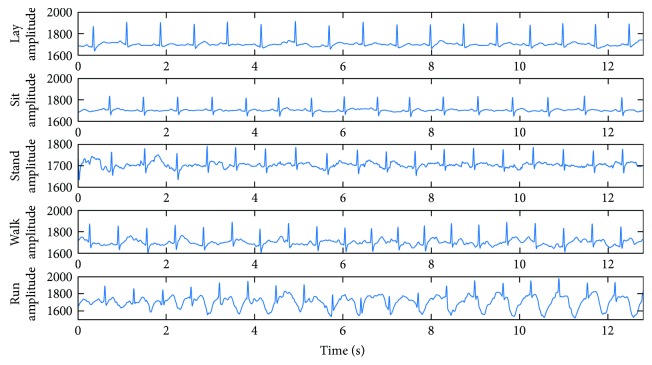
The ECG signals for compression tests.

As shown in Figure 4, the 12.8 s (2560 points) ECG data were used for compression tests, which were sampled by the proposed node in five predefined daily activities, such as lay, sit, stand, walk, and run. The original signal and compressed data were transmitted to the base station, where the ECG signals were reconstructed by the recovery algorithm.

To quantify the compression performance, the percentage root-mean-square difference (PRD) with different normalization [29] is used to quantify the recovered signal quality.

(2) $$\begin{array}{c} PRD=\frac{{\left\|X-Y\right\|}_{2}}{{\left\|X-\bar{X}\right\|}_{2}}\times 100. \end{array}$$

*X* is the original signal, *Y* is the reconstructed signal, and $\bar{X}$ is the mean of *X*. Meanwhile, the parameters *M* and *K*, which closely relate to compression ratio and computational complexity, are adopted to evaluate the compression efficiency.

In the energy consumption tests, the power supply is 3 V, and a 10 Ohm precise resistor is used to transform current to voltage. The DAQ card records the node voltage *V*_node_ and resistor voltage *V*_R_. The card sampled the analog voltages at 5 KHz and then calculated the node's power and energy consumptions in the digital domain. The power consumption *P* is calculated as

(3) $$\begin{array}{c} P=V_{node}\left(\frac{V_{R}}{R}\right), \end{array}$$

where *R* represents the value of the resistor. The energy consumption *E* is given by the summation of the power consumption at a time interval *T*:

(4) $$\begin{array}{c} E=V_{node}\left(\sum_{T}\frac{V_{R}}{R}\right). \end{array}$$

After all experiments, the proposed node was compared with three commercial ECG nodes, such as Shimmer2, ZMP® ECG2, and Zio Patch monitor concerning size, weight, lifetime, and so forth.

## 4. Results

**Figure 5:**
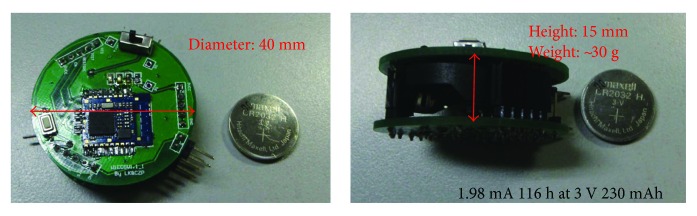
The prototype of single-spot Bluetooth ECG node.

The prototype of proposed single-spot Bluetooth ECG node is shown in Figure 5. The size and weight of proposed node are 40 (diameter, D) × 15 (height, H), and 30 g, respectively.

The comparison experiment was carried out to identify proper recovery algorithm and sparse basis. The PRDs of recovered ECG signals were calculated and reported in Table 1. It is observed that the combination of BSBL [20] and DCT basis achieves the highest signal recovery quality, and the PRDs are less than 3.6%. Besides, the BSBL also beats the recovery algorithms of orthogonal matching pursuit (OMP) [14] and L1 convex optimization [15] under wavelet transform (WT) basis. Since ECG signal is block sparse and correlation structure, BSBL algorithm, which considers such characteristics in the solution of CS, obtained good performance in ECG recovery. The results suggest that the combination of BSBL and DCT basis is a good choice for the base station. The rest experiments are based on BSBL and DCT basis.

Parameters of measurement matrix play an important role in the performance of data CS compression. SBM has three parameters, which are *N*, *M*, and *K*. In this study, *N* is fixed to 512, which means each time the compression algorithm will process segmented 2.56 s ECG signal; due to compression ratio equal to *M*/*N*, parameter *M* takes a significant role in compression efficiency; and it needs *N* × *K* times accumulation to achieve compressed signal. Parameter *K* is a critical measure for computation of compression.

**Figure 6:**
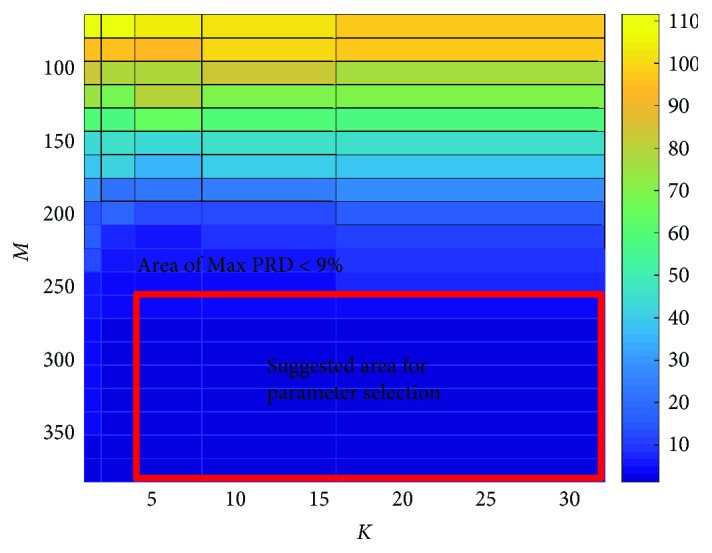
The relationships of signal distortion (PRD), compression efficiency (*M*), and computation complexity (*K*).

To decrease the risk of signal distortion, the maximum PRDs were preserved under *M* and *K* verified from 64 to 384 and 1 to 32, respectively. The relationships of the signal distortion, compression efficiency, and computation complexity are shown in Figure 6. The PRD decreases with the increasing *M* but is insensitive to the change of *K*. Considering the cases with PRD < 9% will not lead diagnostic distortion [29]; a blue marker of PRD = 9% is overlapped in Figure 6 as a benchmark of the accepted recovery area. Meanwhile, considering generalization risk of recovery, the area marked by a solid red rectangle (as shown in Figure 6) is suggested as the parameter selection area, and *M* = 256 and *K* = 4 are the best choice. The computation of the confirmed optimal SBM is 2048 accumulation, which is suitable for the proposed node. It can be concluded that the SBM CS compression is low computation complexity.

**Figure 7:**
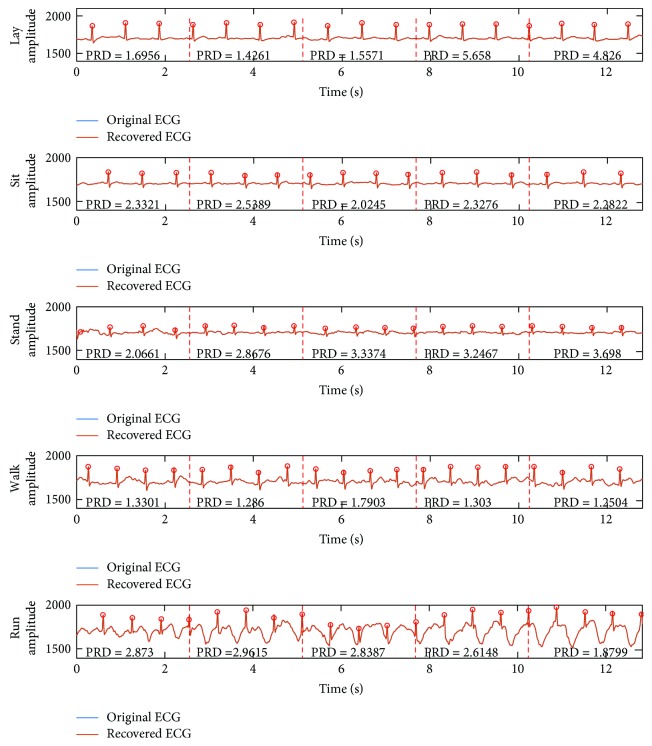
The visual inspection results. The recovery algorithm and sparse bases are BSBL and DCT, respectively: *N* = 512, *M* = 256, and *K* = 4; Pan-Tompkins method [30] was used for *R*-peak detection in the recovery signals. The red dotted lines are the segmented indicators of every frame, and the signal quality of each recovered frame was evaluated by PRD.

The visual inspection of five original and recovered ECG signals is illustrated in Figure 7. It is observed that the PRDs of all records are less than 9%; the proposed system can achieve high-quality ECG signal recovery and guarantee the nondistortion diagnosis. Compared with the length of original signals, half of the data was reduced, which indicates that the proposed method has good potential in energy saving during ECG data wireless transmission. Moreover, the proposed CS compression is nonadaptive. No matter what the original ECG with different rhythms and morphological characteristics was fed in the framework, the same length of compressed data is achieved. Furthermore, all *R*-peaks of recovered ECG signals were detected in Figure 7. It is believed that the proposed system can achieve the recovered signal without diagnosis distortion; the proposed node is qualified for the ambulatory ECG monitoring.

**Figure 8:**
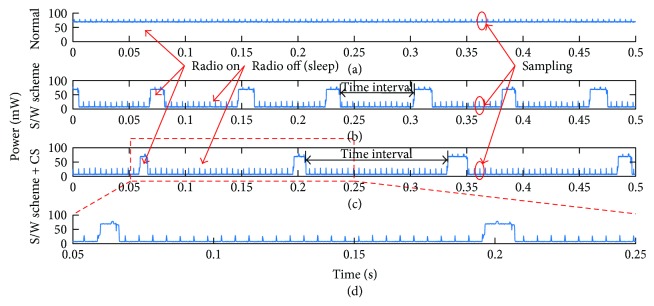
Power traces of the proposed node during different schemes. (a) The normal scheme, no energy saving strategy is used. (b) The S/W scheme. (c) The S/W scheme + CS. (d) The detailed figure of (c). The sampling energy consumption is marked by the red ellipses in (a–c).

**Figure 9:**
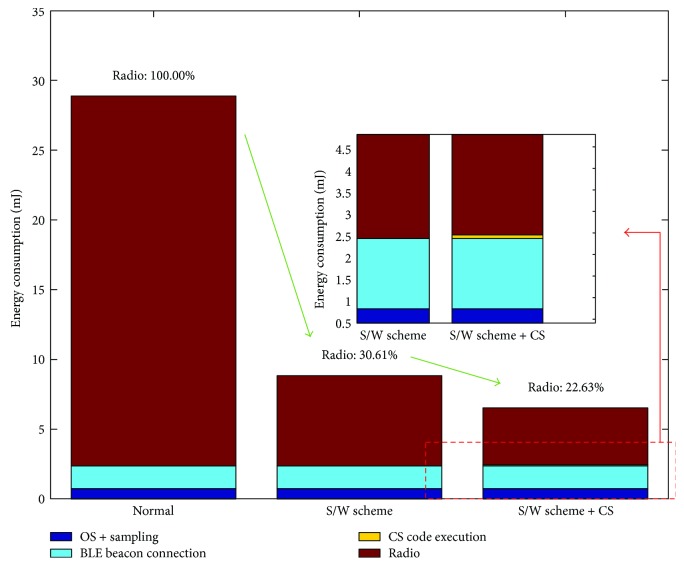
Energy consumption of the proposed node during different schemes.

The power consumptions of the node are demonstrated in Figure 8. It is observed that the power of the normal scheme (Figure 8(a)) holds steady about 70 mW if it ignores the energy consumption of 200 Hz ADC sampling. The node is energy wasting in the normal scheme because the radio is always on; in the S/W scheme, the node periodically sleeps or wakes up, and it turns off or on the radio according to the requirement of the task. The power trace of S/W scheme is like a periodic pulse curve in Figure 8(b). In the last CS + S/W scheme (Figure 8(c)), the time interval of radio off is two times larger than that of S/W scheme, which proves that the CS compression reduces half of the data. The energy consumption of the proposed node is elaborated in Figure 9. The consumed energy in normal, S/W, and CS + SW schemes is 28.87 mJ, 8.85 mJ, and 6.53 mJ, respectively. Assume the energy consumption of radio is 100% in the normal scheme, and it reduces to 30.61% and 22.63% in S/W and CS + SW schemes, respectively. The energy consumption of CS code execution accounts for only 1.3% of the total energy consumption. The results indicate that the proposed node is energy efficient.

The specifics of the proposed node and the comparison with three commercial nodes are reported in Table 2. The proposed node is a light, low-cost, energy-efficient, single-spot wireless ECG node, it can provide real-time ambulatory ECG monitoring, and the lifetime of the node is 116 hours at 3 V battery.

## 5. Discussions

The advantages of the proposed ECG node include energy efficient, low computational complexity compression, real-time, and wireless. By using the ultra-low-power chips, periodic sleep/wake-up strategy, and CS compression, the transmission data was nonadaptive reducing 50% and recovered signal without diagnosis distortion. As indicated in Section 4, sleep/wake-up strategy and CS compression reduce 77.37% radio energy consumption. Meanwhile, the compression algorithm is a low computational complexity. The energy consumption of CS code execution is negligible due to the light computation load of accumulation. Furthermore, the ECG signal can be provided real-time to a user through the proposed system framework.

**Figure 10:**
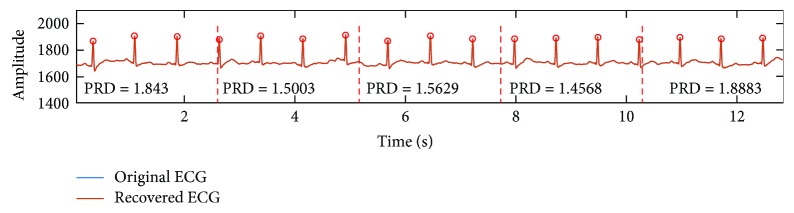
The recovered signal with 10 points delay.

In the first subfigure of Figure 7, it can be found that the morphological similarity of ECG signal is high and the noise level of ECG signal is stable, but the PRDs are suddenly increased. ECG signal is typically characterized by localized information where essential and valuable diagnostic information is concentrated at the small interval and high-amplitude QRS complex. CS is a sampling method following the “information rate.” The whole QRS complex is segmented into two parts which leads to information loss that leads to distortion. The 10-point delayed recovered ECG is shown in Figure 10, and it is observed that there is no great variation of PRD.

The distortion caused by QRS complex location is the major shortcoming in our proposed ECG system, which is expected to be solved by the information-enhanced sparse binary matrix [32].

## 6. Conclusion

CS is capable of achieving high compression ratios with low computational and memory requirements, making it suitable for being used in wireless ECG nodes. A digital compressed sensing-based single-spot Bluetooth ECG node was proposed in this study. The node was optimized for hardware, sleep/wake-up strategy, and CS compression and achieved good performance in energy saving. The proposed node reduces 77.37% radio energy consumption under compressed half of the data. The SBM-based compression algorithm is a low computational complexity and nonadaptive, and the energy consumption of CS code execution is negligible. The recovered signals are essentially undistorted. Thus, it can be concluded that the proposed node can reduce the energy requirement in transmitting ECG data and retaining the information content for diagnosis. The comparison with other ECG node shows that the advantages of the proposed node include light, low-cost, single-spot, real-time and wireless.

## Figures and Tables

**Table 1:** PRD of the recovered signal under different sparsity bases and recovery algorithms, *N* = 512, *M* = 256, *K* = 4.

Experimental conditions	PRD (%)				
	Lay	Sit	Stand	Walk	Run
BSBL + WT [20]	6.35	6.20	9.58	5.66	7.23
BSBL + DCT [20]	**3.54**	**2.28**	**2.91**	**1.39**	**2.52**
OMP + WT [14]	7.69	10.35	21.34	12.01	11.85
L1 + WT [15]	10.68	11.67	20.05	16.30	11.80

**Table 2:** Comparison of the proposed node with commercial ones.

	Proposed node	Shimmer2	ZMP ECG2	Zio Patch [31]
Size (mm)	40 (D) × 15(H)	53 × 32 × 23	44 × 41 × 9.34	123 × 53 × 10.7
Weight (g)	30	32	15	34
Current				
OS + sampling	0.23 mA	0.1 mA (only OS)	~1.35 mA	—
Wireless	1.98 mA at 200 Hz	~20 mA	~3.00 mA	No radio
Deep sleep	5.2 *μ*A	0.14 mA	~4 *μ*A	—
Lifetime	116 h at 3 V 230 mAh	~24 h at 3.7 V 450 mAh	72 h at 3.3 V 230 mAh	<14 day
Location	Single-spot	Traditional three lead	Single-spot	Single-spot
Price (RMB)	<400 (hardware cost)	>2800	>20000	—

Shimmer2: http://www.shimmersensing.com/; ZMP ECG2: https://www.zmp.co.jp; and Zio Patch monitor: http://irhythmtech.com/.
